# Case Report: Lung adenocarcinoma associated with germline *ERCC2* frameshift mutation

**DOI:** 10.3389/fonc.2023.1177942

**Published:** 2023-05-08

**Authors:** Lili Liu, Jia Cui, Siye Liu, Evenki Pan, Limin Sun

**Affiliations:** ^1^ Department of Medical Oncology, The Second Affiliated Hospital of Dalian Medical University, Dalian, China; ^2^ Department of Medical, Nanjing Geneseeq Technology Inc., Nanjing, Jiangsu, China

**Keywords:** germline mutation, lung adenocarcinoma, ERCC2 frameshift mutation, family history, next-generation sequencing

## Abstract

Family history is an established risk factor for lung cancer. Previous studies have found that germline genetic alterations, such as those in *EGFR*, *BRCA1*, *BRCA2*, *CHEK2*, *CDKN2A*, *HER2*, *MET*, *NBN*, *PARK2*, *RET*, *TERT*, *TP53*, and *YAP1*, are associated with an increased risk of developing lung cancer. The study reports the first of a lung adenocarcinoma proband with germline *ERCC2* frameshift mutation c.1849dup (p. A617Gfs*32). Her family cancer history review demonstrated that her two healthy sisters, a brother with lung cancer, and three healthy cousins were positive for *ERCC2* frameshift mutation, which might contribute to increased cancer risk. Our study highlights the necessity of performing comprehensive genomic profiling in discovering rare genetic alterations, early cancer screening, and monitoring for patients with family cancer history.

## Introduction

The germline genetic alterations in multiple genes have demonstrated significant risks for several cancers such as breast, colorectal, melanoma, and ovarian. Although cigarette smoking is considered to be the predominant risk factor for most lung cancers (1), multiple studies have revealed that many lung cancer patients present a family clustered pattern. Moreover, probands of family cases had a significantly increased risk as never-smokers (2). Previous studies have found that germline mutations of *EGFR*, *BRCA1*, *BRCA2*, *CHEK2*, *CDKN2A*, *HER2*, *MET*, *NBN*, *PARK2*, *RET*, *TERT*, *TP53*, and *YAP1* were associated with lung cancer risk (3), among which *EGFR*, especially *EGFR* T790M, was by far most frequently reported genetic alterations (4), mainly because the use of tyrosine kinase inhibitor (TKI) is closely related to *EGFR* mutations.

Germline genetic alterations in DNA repair genes are a common cause of hereditary cancer predisposition. *ERCC2*, an emerging cancer gene, plays a main role in the process of nucleotide excision repair (NER). Mutations in *ERCC1–5*, encoding the core NER, lead to inherited syndromes associated with increased cancer risk (5). However, cancer risk associated with defects in genes that regulate NER is less well understood, and there are no United States Food and Drug Administration (FDA)-approved targeted therapies for patients with germline or somatic alterations in NER genes (6). Here, we first report a lung adenocarcinoma proband with germline *ERCC2* frameshift mutation c.1849dup (p. A617Gfs*32).

## Case presentation

**Figure 1:**
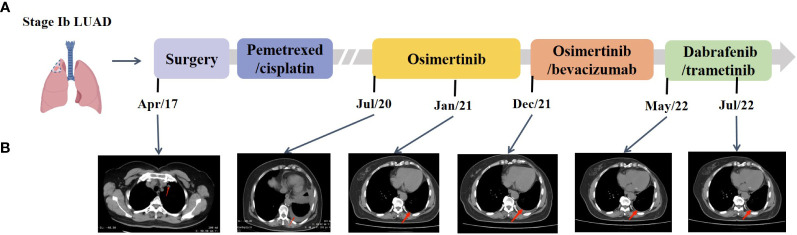
Representative clinical images during the treatment course. **(A)** Disease timeline showed the various treatment received by the patient and her clinical response. LUAD, lung adenocarcinoma. **(B)** Chest CT scans showed the disease progression under treatment.

**Figure 2:**
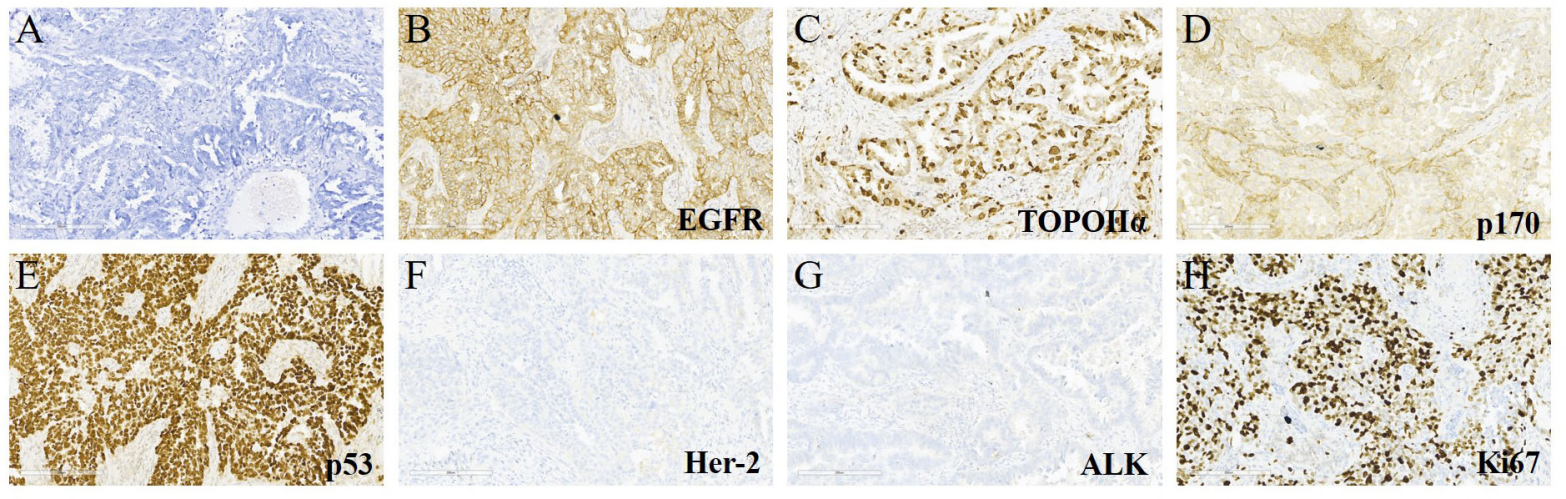
Hematoxylin & eosin (H&E) and immunohistochemical (IHC) staining for the lung tumor tissues. **(A)** H&E staining (×200) of the lung cancer. **(B–H)** IHC examinations (×200) of the resected tumor tissues were positive for epidermal growth factor receptor (EGFR), topoisomerase IIα (TOPOIIα), 170-kDa protein (p170), and p53 and negative for human epidermal growth factor receptor 2 (Her-2) and ALK. The Ki67 index was 60%.

**Figure 3:**
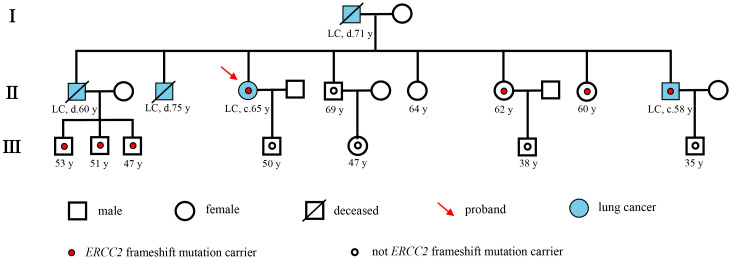
Pedigree describing the patient’s personal and family history of cancer. Squares and circles denote male and female, respectively. Roman numerals indicate generations. The proband was marked with a red arrow. A small red circle indicates which family members were tested and found to carry *ERCC2* frameshift mutation. A small hollow circle indicates which family members were tested and found not to carry *ERCC2* frameshift mutation.

A 65-year-old female non-smoker had suffered from a cough for several days in April 2017. The integrated positron emission tomography (PET) and computed tomography (CT) revealed a mass (1.7 × 1.7 × 1.5 cm^3^) in the upper lobe of the left lung, which was surgically removed (**Figure 1B**). Immunohistochemistry (IHC) examinations of the resected tumor tissues were positive for epidermal growth factor receptor (EGFR), topoisomerase IIα (TOPOIIα), 170-kDa protein (p170), and p53 and negative for human epidermal growth factor receptor 2 (Her-2) and ALK. Ki67 labeling in the tumor cells was 60% (**Figure 2**). Based on the histological and IHC results, the patient was diagnosed with stage Ib invasive adenocarcinoma of the left upper lobe of the lung (pT2aN0M0) (**Figure 1A**). Four cycles of postoperative adjuvant chemotherapy with pemetrexed and carboplatin were administrated. In July 2020, the disease progressed with pleural metastases (**Figure 1B**). To identify a more efficient therapeutic strategy, hydrothorax samples were subjected to targeted next-generation sequencing (NGS) of 580 cancer-related genes. Genomic profiling observed *EGFR* L858R with a mutant allelic frequency (MAF) of 17.35% and splicing mutation in intron 6 of *RET* gene (c.1263 + 2T>C, MAF = 22.21%). The patient then received osimertinib (80 mg qd), a third-generation EGFR TKI, and achieved an initial partial response (PR) with sustained response ongoing for 17 months (**Figure 1B**). In December 2021, clustered tumor cells were identified in the pleural puncture samples. As shown in **Table 1**, the follow-up targeted genomic testing with 425 cancer-related genes (Nanjing Geneseeq Technology Inc., Nanjing, China) indicated *EGFR* (L858R), *EGFR* (L718Q), *BRAF* (V600E), *TP53* (R273H), *RET* (c.1263 + 2T>C) mutations, and germline *ERCC2* frameshift mutation c.1849dup (p. A617Gfs*32) in hydrothorax samples. A combined treatment of bevacizumab and osimertinib was administrated in December 2021. We investigated her family history and collected blood biopsies from her 11 relatives, 10 of whom have no symptoms of cancer up to date, and one was diagnosed with lung cancer at 58 years old. Family history showed her father (age 71) and two elder brothers (age 60 and 75) died of lung cancer. Genetic testing revealed another six carriers of germline *ERCC2* frameshift mutation c.1849dup (p. A617Gfs*32) in addition to the proband, her two healthy sisters, a brother with lung cancer, and three healthy cousins (**Figure 3**). Five months later, the disease progressed with the detection of increased AF *BRAF* (V600E) mutation (**Table 1**). The patient then received dabrafenib (150 mg bid) plus trametinib (2 mg qd) in May 2022 and achieved stable disease (SD) with sustained response ongoing for 2 months (**Figure 1B**).

**Table 1:** Genomic alterations by targeted NGS detected in hydrothorax samples during the disease course.

Gene	Alterations	Before treatment		
		Osimertinib	Bevacizumab + osimertinib	Dabrafenib + trametinib
*ERCC2*	p. A617Gfs*32	–	Germline	
*EGFR*	p. L858R	17.35%	31.0%	38.7%
*RET*	c.1263 + 2T>C	22.21%	32.7%	40.4%
*EGFR*	p. L718Q	–	2.5%	4.9%
*BRAF*	p. V600E		1.3%	7.1%
*TP53*	p. R273H	–	28.4	38.8%
*TSC1*	p. K745Efs*9	–	0.5%	5.2%
*TSC2*	p. E1679Afs*149	–	0.6%	0.3%
*DAXX*	p. E657K	–	7.9%	9.1%
*NYRK3*	p. S143L	–	0.4%	–
*PTK2*	p. Y429*	–	10.5%	18.0%
*WT1*	p. P9T	–	9.6%	12.4%

Mutation was shown as mutant allele frequency.

-, not detected; NGS, next-generation sequencing.

## Discussion

There is increasing evidence about the presence of germline pathogenic variants in lung cancer patients. Germline susceptibility loci of multiple genes in lung cancer patients were reported to be associated with lung cancer risk, including *EGFR*, *BRCA1*, *BRCA2*, *CHEK2*, *CDKN2A*, *HER2*, *MET*, *NBN*, *PARK2*, *RET*, *TERT*, *TP53*, and *YAP1.* Due to the selection of different populations and different target genes, the prevalence of germline genetic alterations found in various studies was reported to be different (3).

In this report, we first described a patient with lung adenocarcinoma harboring germline *ERCC2* frameshift mutation c.1849dup (p. A617Gfs*32). When we investigated her family history, we found that her four relatives had lung cancer and that six relatives carried the same *ERCC2* germline frameshift mutation. Especially, her brother who died of lung cancer (II-1) was an obligate carrier of the mutation because all three of his children were carriers, which reinforces the link of *ERCC2* germline frameshift mutation to the risk of lung cancer. *ERCC2*, an emerging cancer gene, is the ATP-dependent DNA helicase, which is a part of the transcription factor IIH (TFIIH) complex involving RNA polymerase II-mediated transcription and associated with the process of NER. After recognition of DNA damage by the NER sensor, the repair is performed through the unwinding of the DNA at the damage site by helicases ERCC2 and ERCC3, incision by endonucleases ERCC1/4/5, and subsequent error-free gap filling and ligation. The *ERCC2* frameshift mutation c.1849dup (p. A617Gfs*32) in the exon 20 caused a tandem repeat of the 1849th base, resulting in the insertion of a sequence of identical bases. This led to a frameshift starting from the 617th amino acid, causing premature termination codons and producing a truncated protein. This may lead to the deactivation of the *ERCC2* gene, resulting in the production of structurally abnormal ERCC2 protein, which loses its ability to repair damaged DNA and may increase the risk of developing cancer.

Numerous studies have suggested that common single-nucleotide polymorphisms (SNPs) in *ERCC2* may reduce normal cellular NER capability and affect melanoma, bladder, and lung cancer risk non-smokers (6–8). Somatic *ERCC2* variations are found at a low frequency in lung cancer and have been reported to correlate with increased cisplatin sensitivity (9). In addition, germline mutations in DNA repair genes can be associated with increased cancer risk and act as an important predictive biomarker in several clinical contexts that can be helpful for early cancer detection. However, lung cancer risk associated with germline mutations in *ERCC2* is less well understood, and currently, there are no FDA-approved targeted therapies, thus providing further motivation to characterize the functional impact of *ERCC2* mutations in lung cancer patients. In our patient’s family, in the four germline *ERCC2* frameshift mutation carriers who have not shown any symptoms, routine medical examinations and early cancer screening should be performed.

The limitation of the single-case presentation in this study should also be noted. Thus, the association between the *ERCC2* frameshift mutation *c.1849dup* (p. A617Gfs*32) and the lung cancer risk is not so easy to determine; however, additional pre-clinical studies and additional clinical evidence are needed.

## Conclusion

We reported the first case of a germline *ERCC2*-mutated lung adenocarcinoma with confirmed family history. The report highlights the importance of performing comprehensive genomic profiling in discovering rare gene genetic alterations, as well as early cancer screening and ongoing monitoring for individuals with a family history of cancer. We also revealed that the heredity of *ERCC2* frameshift mutation may increase the likelihood of developing lung cancer.

## Data availability statement

The raw data supporting the conclusions of this article will be made available by the authors, without undue reservation.

## Ethics statement

This research was approved by the Ethics Committee of The Second Hospital of Dalian Medical University. Written informed consent to publish the clinical details and images were obtained from the patient.

## Author contributions

All authors contributed to data analysis and drafting or revising of the manuscript. All authors agreed on the journal to which the article is submitted, provided final approval of the manuscript version to be published, and agreed to be accountable for all aspects of the study.
